# Antitumor Activity of Auger Electron Emitter ^111^In Delivered by Modular Nanotransporter for Treatment of Bladder Cancer With EGFR Overexpression

**DOI:** 10.3389/fphar.2018.01331

**Published:** 2018-11-19

**Authors:** Andrey A. Rosenkranz, Tatiana A. Slastnikova, Tatiana A. Karmakova, Maria S. Vorontsova, Natalia B. Morozova, Vasiliy M. Petriev, Alexey S. Abrosimov, Yuri V. Khramtsov, Tatiana N. Lupanova, Alexey V. Ulasov, Raisa I. Yakubovskaya, Georgii P. Georgiev, Alexander S. Sobolev

**Affiliations:** ^1^Institute of Gene Biology, Russian Academy of Sciences, Moscow, Russia; ^2^Faculty of Biology, Lomonosov Moscow State University, Moscow, Russia; ^3^National Medical Research Radiology Center of the Ministry of Healthcare of the Russian Federation, Moscow, Russia; ^4^National Research Nuclear University MEPhI (Moscow Engineering Physics Institute), Moscow, Russia

**Keywords:** modular nanotransporters, drug delivery, intracellular transport, bladder cancer, radionuclide therapy, Auger electron emitter, indium-111

## Abstract

Gamma-ray emitting ^111^In, which is extensively used for imaging, is also a source of short-range Auger electrons (AE). While exhibiting negligible effect outside cells, these AE become highly toxic near DNA within the cell nucleus. Therefore, these radionuclides can be used as a therapeutic anticancer agent if delivered precisely into the nuclei of tumor target cells. Modular nanotransporters (MNTs) designed to provide receptor-targeted delivery of short-range therapeutic cargoes into the nuclei of target cells are perspective candidates for specific intracellular delivery of AE emitters. The objective of this study was to evaluate the *in vitro* and *in vivo* efficacy of ^111^In attached MNTs to kill human bladder cancer cells overexpressing epidermal growth factor receptor (EGFR). The cytotoxicity of ^111^In delivered by the EGFR-targeted MNT (^111^In-MNT) was greatly enhanced on EJ-, HT-1376-, and 5637-expressing EGFR bladder cancer cell lines compared with ^111^In non-targeted control. *In vivo* microSPECT/CT imaging and antitumor efficacy studies revealed prolonged intratumoral retention of ^111^In-MNT with *t*½ = 4.1 ± 0.5 days as well as significant dose-dependent tumor growth delay (up to 90% growth inhibition) after local infusion of ^111^In-MNT in EJ xenograft-bearing mice.

## Introduction

The promising agents for cancer therapy are aimed at molecular targets that most differentiate cancer cells from normal cells. Many of these molecular targets including the epidermal growth factor receptor (EGFR) family are tyrosine kinase receptors that are often involved in malignancy (Krause and Van Etten, 2005; Li et al., 2012). Therefore, it is not surprising that there are a significant number of drugs acting on these targets that are either approved for cancer treatment or undergoing clinical trials (Fauvel and Yasri, 2014). Many of these targets are also considered as a basis for the development of cancer diagnostics tools, in particular for different types of emission tomography (Corcoran and Hanson, 2014). Emission tomography is now widely used in medicine, including in cancer diagnostics (van Essen et al., 2014; Yankeelov et al., 2014), with tens of millions of scans carried out worldwide annually (Benard et al., 2014). Gamma-ray emitting ^99m^Tc, ^111^In, ^123^I, and ^67^Ga extensively used for imaging are also sources of low-energy Auger electrons (AE), which have a short-distance distribution range (Volkert et al., 1991). These electrons exert negligible effect outside cells. However, they become highly toxic in the vicinity of DNA within the cell nucleus (Volkert et al., 1991; Buchegger et al., 2006; Kassis, 2008).

^111^In, emitting 1.84 gamma ray photons per decay, with a convenient for SPECT energy 171 and 245 keV is the radioisotope which can be considered as a theranostic agent upon attachment to the appropriate targeting biomolecule (Levine and Krenning, 2017; Pencharz et al., 2018). In this way beginning with Krenning’s work (Krenning et al., 1994) it had been being demonstrated that using therapeutic doses of the radiopharmaceutical [^111^In-DTPA]-D-Phe^1^-octreotide (^111^In-octreotide) for treatment of neuroendocrine tumors does not lead to severe side effects (Janson et al., 1999; Caplin et al., 2000; Anthony et al., 2002; Valkema et al., 2002; Buscombe et al., 2003; Nguyen et al., 2004; Kong et al., 2009; Ozkan et al., 2011). More than 140 cases of therapy using this radiopharmaceutical are analyzed in these papers. In those studies where the comparative dosimetric estimations were carried out the mean cumulative tumor absorbed doses ranged from 3.7 mGy/MBq (Forster et al., 2001) to 9.4 mGy/MBq (Helisch et al., 2004) compared to the mean cumulative liver absorbed doses of 0.6 and 0.4 mGy/MBq, respectively. Locoregional intra-arterial administration of ^111^In-octreotide via the hepatic artery (Limouris et al., 2008; Pool et al., 2014) can increase the ratio of absorbed dose of unresectable liver metastases (10.8 mGy/MBq) to the normal liver absorbed dose (0.14 mGy/MBq) (Limouris et al., 2008).

Internalization as well as nuclear localization of ^111^In-octreotide had been described, though the percentage entering the nucleus was generally small (Andersson et al., 1996; Janson et al., 2000). A direct Auger radiation effect on DNA was therefore expected (Buchegger et al., 2006). Therapeutic effects after injections of the radiopharmaceutical consisted mostly in stabilization of tumor growth, partial remissions remaining scarce (Buchegger et al., 2006). Apparently, greater intranuclear accumulation should lead to a more pronounced outcome. The nuclear localization of another radiopharmaceutical ^111^In-DTPA-human EGF was also insufficient for the noticeable therapeutic effect (Vallis et al., 2014). Our more complex constructs, the modular nanotransporter (MNT), are able to effectively deliver substances into the cell nuclei transporting more than half of intracellular AE emitters during the first hours of incubation (Slastnikova et al., 2012a; Koumarianou et al., 2014). We consider MNT as a multifunctional delivery platform for transport of AE emitters into the nuclei of cancer cells (Sobolev et al., 2016; Sobolev, 2018). This platform is now represented by several MNTs that are promising tools for delivery of anticancer drugs into nuclei of cancer cells with increased expression of internalizable surface receptors (Slastnikova et al., 2012a,b, 2017a,b; Koumarianou et al., 2014; Rosenkranz et al., 2017; Sobolev, 2018) in this journal issue. The MNTs consist of several functional polypeptide modules providing targeted intracellular transport including specific binding to and endocytosis by a target cancer cell, escape from endosomes into hyaloplasm, and subsequent active transport into the cell nucleus via the nuclear pore (Rosenkranz et al., 2003; Sobolev, 2008; Sobolev et al., 2016). The *in vitro* and *in vivo* ability of MNTs to reach the nuclei of target cells has been specifically demonstrated previously by confocal laser microscopy and nuclear isolation (Rosenkranz et al., 2003; Gilyazova et al., 2006; Slastnikova et al., 2012a,b, 2017a; Koumarianou et al., 2014). The cell specificity of MNTs is ensured by a ligand module that binds to surface receptors overexpressed on cancer cells. The MNT aimed at EGFR, with epidermal growth factor (EGF) serving as a ligand module, is attractive for the clinic because EGFR expression is enhanced in many malignances, including lung cancer (Hirsch et al., 2003), head and neck cancer (Kalyankrishna and Grandis, 2006), bladder transitional cell carcinomas (Chow et al., 2001), etc. In this study, we evaluated the therapeutic potential and pharmacokinetics profile of the AE emitter ^111^In delivered by EGFR-targeted MNTs to human bladder tumor cells *in vitro* and *in vivo*.

## Materials and Methods

### Cell Lines

Human urinary bladder cancer cell lines 5637, HT-1376, and SCaBER (all from the American Type Culture Collection, ATCC, Manassas, VA, United States) and EJ, T24, and, RT4 (from the Russian Collection of Cell Cultures, Moscow) as well as human epidermoid carcinoma cells (from ATCC) were maintained according to the specifications of the collections.

### Labeling of Epidermal Growth Factor With ^125^I

Human EGF (Sigma Chemicals, St. Louis, MO, United States) was labeled with ^125^I (Khlopin Radium Institute, Russia) using Iodogen (1,3,4,6-tetrachloro-3α,6α-diphenylglycoluril, Sigma, United States). For labeling, 10 μg of the protein and 20–40 MBq of radioiodide in 0.05 M sodium borate buffer (pH 8.5) were incubated in glass vials coated with 10 μg of Iodogen for 15 min at room temperature. The reaction was terminated by addition of tyrosine to final concentration 5 mM. Radioiodinated EGF was purified by gel filtration through a PD-10 column (GE Healthcare Life Science, Great Britain) that was eluted with phosphate-buffered saline (pH 7.5). The yield for the radioconjugation reaction was 70–80% and the initial specific activity of ^125^I-iodoEGF ranging from 2.2 to 3.1 GBq/mg of protein.

### Binding Assays

The EGFR status of cells was estimated using ^125^I-iodoEGF. In brief, the cells were seeded at a density of 2–4 × 10^5^ cells per well in 24-well plates. After 36 h, serial dilutions of ^125^I-iodoEGF in 200 μl were added into the wells, and the cells were incubated with ligands for 18 h at 4°C in Dulbecco’s Modified Eagle Medium without sodium bicarbonate but supplemented with 20 mg/ml of bovine serum albumin (BSA) and 20 mM 4-(2-hydroxyethyl)-1-piperazineethanesulfonic acid (HEPES) (pH 7.5). The addition of 1.5 μM of non-labeled EGF was used to measure non-specific binding. The cells were washed three times with the same ice-cold medium on ice, lysed in 0.5 M NaOH for 30 min, and the radioactivity associated with the cell lysates was measured in a Ria Gamma 1271 γ-counter (LKB, Sweden). The affinity constant of ^125^I-iodoEGF binding to EGFR and concentration of binding sites for ^125^I-iodoEGF was calculated by non-linear regression of binding data (one-site binding model). The affinity constants and maximum binding values were calculated. The experiments were carried out in triplicate, and error bars on graphs represent the SEM.

### Modular Nanotransporters

The MNT DTox-HMP-NLS-EGF consisting of human EGF as a ligand module, diphtheria toxin translocation domain (DTox) as an endosomolytic module, optimized nuclear localization sequence of SV-40 large T-antigen (NLS) as a module providing transport into the nucleus, and *Escherichia coli* hemoglobin-like protein (HMP) as a carrier module was produced as described previously (Gilyazova et al., 2006; Slastnikova et al., 2017b). Briefly, the MNT was expressed in an *E. coli* by the addition of isopropyl-β-d-1-thiogalactopyranoside to a final concentration 0.2 mM at 18°C for 2 h. The suspension was centrifuged at 10,000 rpm with a JA-10 rotor (Beckman Coulter, Brea, CA, United States) at 4°C for 30 min, and the pellets were lysed in ice-cold 50 mM sodium phosphate, 300 mM NaCl, pH 8.0, 1 mM phenylmethylsulfonyl fluoride, 5 mg/ml lysozyme, and 0.5% Triton X-100. The lysate was clarified by centrifugation at 18,000 rpm with the JA-20 rotor at 4°C for 30 min. Then the supernatant was loaded onto an Ni-NTA agarose column (Qiagen NV, Venlo, Netherlands), washed with 50 mM sodium phosphate, 300 mM NaCl, 20 mM imidazole, pH 8.0, 0.5% Triton X-100, and 1% glycerol, followed by 50 mM sodium phosphate, 300 mM NaCl, 20 mM imidazole, pH 8.0. The MNT was eluted with 50 mM sodium phosphate, 300 mM NaCl, 700 mM imidazole, pH 8.0, and dialyzed against 10 mM sodium phosphate, 150 mM NaCl, pH 7.4.

### Labeling of the MNT With ^111^In

To permit subsequent ^111^In labeling, the chelator p-SCN-Bn-NOTA (Macrocyclics, Plano, United States) [log KIn(III) NOTA = 26.2 (Sun et al., 2000)] was conjugated to the MNT DTox-HMP-NLS-EGF according to a recently published protocol (Slastnikova et al., 2017b). All buffers for chelator conjugation and labeling procedures used were passed through Chelex-100 resin (200–400 mesh, Bio-Rad) to minimize adventitious heavy metal ion contamination. Briefly, the MNT was incubated with 10-fold molar excess of the chelator in sodium carbonate buffer (Koumarianou et al., 2014) with 5 mM EDTA, pH 8.6, for 20 h at RT with final concentrations of MNT ≥1.5 mg/ml. The chelator-MNT conjugate was concentrated and separated from excess chelator by five cycles of ultrafiltration using an Amicon Ultracel-30K. During this process, the conjugation buffer was gradually replaced with 10 mM HEPES, 15 mM NaCl, pH 7.4. The resulting NOTA-MNT was freeze-dried in aliquots and stored at 4°C. Control MNT DTox-HMP-NLS lacking any ligand module was modified with NOTA in the same manner. The chelator: MNT molar ratio was determined by MALDI-MS performed on a MALDI TOF/TOF Mass Spectrometer (Shimadzu, Kyoto, Japan). The average number of chelators attached to the MNT molecule was 2.5 ± 0.2. ^111^In was conjugated to the MNT in solution containing 80 mM HEPES, 6 mM citrate, 0.02% SDS, pH 4.5 for 1 h at 37°C, and then the reaction was stopped by addition of EDTA to concentration 0.4 mM. Finally, the solution was neutralized with NaOH. The initial specific radioactivity of ^111^In-NOTA-DTox-HMP-NLS-EGF obtained using this protocol was 2.7 GBq/mg. As a control for cytotoxicity and *in vivo* experiments, the initial ^111^In solution was treated following the same procedures except that the NOTA-MNT was omitted in the reaction mixture. Radiochemical yields ^111^In-NOTA-MNT were assessed by Laemmli SDS–PAGE using Mini-Protean TGX Any kDa gels (Bio-Rad, Hercules, CA, United States) with subsequent detection of radioactivity on a Storm 865 phosphorimager (GE Healthcare, Sweden). The images were analyzed using ImageQuant TL 5.0 software (Bio-Rad). Due to high (96.5 ± 0.1%) radiochemical labeling yields, there was no need for any subsequent purification of the ^111^In-labeled MNT.

### Cytotoxicity Studies

Cells were seeded in 24-well plates (2.5 × 10^4^ cells/well). Two days later the medium was refreshed, and dilutions of ^111^In-NOTA-DTox-HMP-NLS-EGF (0–25 MBq/ml; 0–11 μg/ml), or ^111^In-NOTA-DTox-HMP-NLS (0–25 MBq/ml), or the control ^111^In solution containing 0–25 MBq/ml were added. The cells were incubated for 48 h in a humidified atmosphere at 37°C under 5% CO_2_. Then medium containing unbound radioactivity was removed, and the cells were washed, trypsinized, harvested, and resuspended in 1 ml of fresh medium. The cells were seeded for colony-forming assay in 25-cm^2^ flasks (2000 cells per flask) in DMEM/F12 medium supplemented with 10% CFS. After 6–11 days, the colonies were stained with Crystal Violet and counted.

### Animal Studies

The experiments were performed on 8- to 9-week-old female Balb/c *nu/nu* mice weighing 17–20 g (Institute of Bioorganic Chemistry Regional Affiliate, Russian Academy of Science, Pushchino, Russia). The animals were maintained under specific pathogen-free conditions and were given *ad libitum* access to food and water. The experimental protocol was approved by the Institute Commission for Animals and was performed in accordance with the National Institutes of Health guide for the care and use of laboratory animals. EJ tumors were established in nude mice by subcutaneous injection of 5 × 10^6^ cells suspended in 100 μl of serum-free medium into the back flank region. Single-photon emission computed tomography (SPECT) combined with conventional computed tomography X-ray scanner (CT) imaging and therapy studies were initiated 14 days after tumor inoculation.

### SPECT/CT Imaging

For SPECT/CT imaging, EJ xenograft-bearing mice were anesthetized with 0.8–1.8% isoflurane in air; tumor-bearing mice (*n* = 3 per group) received intratumoral injections of 15 MBq of either ^111^In-NOTA-DTox-HMP-NLS-EGF or control ^111^In in Hanks solution in a volume equal to half of the tumor volume. Whole body imaging was performed on a U-SPECT-II/CT scanner (MILabs, Utrecht, Netherlands) beginning immediately after injection and continuing for 5 × 10-min frames using a 1.0-mm-diameter pinhole collimator with subsequent immediate whole-animal CT acquisition. For high resolution tumor imaging, a 0.35-mm-diameter pinhole collimator was used. Additional SPECT/CT imaging was performed during the subsequent days (3–5 frames × 10 min). Images were reconstructed using U-SPECT-Rec2.34b software from the manufacturer of the instrument, followed by coregistration of SPECT images to the corresponding CT images. Quantitative analysis of images after 3D-reconstruction was performed using PMOD 3.4 software (PMOD Technologies Ltd., Switzerland).

The radioactivity concentration in tumor and other organs or tissues was determined by selecting a few spheres within the organ or tissue of interest followed by division of the summarized signal within each sphere by its volume.

### Therapy Studies

For therapy studies, EJ xenograft-bearing mice (*n* = 3–5 per group) were intratumorally injected with 2.3, 4.6, or 9.2 MBq of ^111^In-MNT (1, 2, or 4 μg of MNT, respectively) in volume 0.04-ml per mouse. Mice of control groups (*n* = 5 per group) were injected with saline or 4 μg of MNT or 9.2 MBq of ^111^In in the same volume. Tumor volume was calculated using the ellipsoid formula (Tomayko and Reynolds, 1989). The efficacy of the treatment was monitored using tumor growth inhibition index (Hather et al., 2014) defined as: [1-(mean volume of treated tumors)/(mean volume of control tumors)] × 100%.

The animals were sacrificed on the 33rd day from treatment and autopsy was performed. The radioactivity of tumor tissues and normal tissues from the contra-lateral flank were measured using a Wizard 2480 automatic gamma counter (Perkin Elmer/Wallac, Finland).

### Pharmacokinetic and Tissue Distribution Experiments

EJ xenograft-bearing mice (*n* = 3 per group) were intratumorally injected with 73 kBq of ^111^In-MNT in volume 0.04 ml per mouse. At 5 min and 3, 24, 48, and 120 h post-injection, mice were sacrificed. The blood, bladder, kidneys, liver, spleen, bone, lungs, heart, intestine, stomach, muscle, and tumors were harvested, weighed, and measured using the Wizard 2480 automatic gamma counter. The tissue radioactivity was expressed as the percentage of injected dose (%ID) per gram of tissue (%ID/g). The data were used for estimation of the peak blood concentration (C_max_, %ID/g), time to reach C_max_ (T_max_), elimination half-life (T½), and the area under the curve (AUC_t_, %ID/g⋅h).

### Histological Analysis

Necropsy samples (tumor, popliteal, and axillary regional lymph nodes), which were obtained from animals at autopsy on the 20th (*n* = 3) and 40th (*n* = 5) day after tumor cell inoculation, were fixed in 10% neutral buffered formalin saline and embedded in paraffin. Serial 4-μm sections were cut from the paraffin blocks, stained with hematoxylin and eosin according to standard procedures, and embedded into Canadian balsam.

### Immunohistochemical Staining

EGFR immunohistochemistry was performed on 4-μm-thick formalin-fixed, paraffin-embedded tissue sections. After deparaffinization and antigen retrieval procedure at 95°C in 1 mM EDTA, PH 8.0, the sections were incubated with 1% H_2_O_2_ in saline to inactivate endogenous peroxidase and then blocked for 30 min in 1% BSA in saline. Primary monoclonal rabbit anti-EGFR antibody (D38B1; Cell Signaling Technology, Danvers, MA, United States) was diluted 1: 50 in blocking buffer and incubated on sections overnight. Secondary biotinylated anti-rabbit polyclonal antibody (Santa Cruz Biotechnology) was used in recommended dilution and incubated on sections for 1 h. Horseradish-labeled streptavidin (Santa Cruz Biotechnology) and DAB+ Liquid Chromogenic Substrate (1: 100; DAKO, Denmark) were used for chromogenic reaction. Slides were counterstained with hematoxylin and mounted into Canadian balsam.

### Statistics

The data were analyzed using GraphPad Prism 6 software (GraphPad Software Inc., San Diego, CA, United States). Data on the plots represent mean values, with bars indicating the standard error of the mean of 3–6 repetitive values.

## Results

### EGFR Expression on Bladder Cancer Cell Lines

**FIGURE 1:**
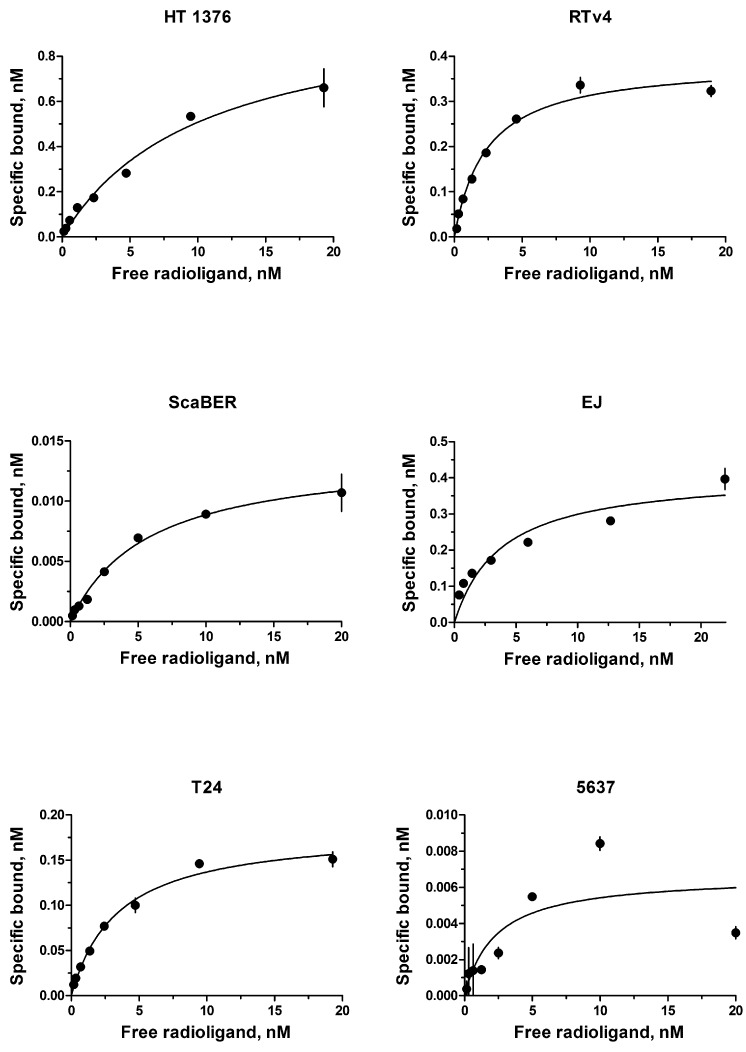
Radioligand analysis of ^125^I-iodoEGF-binding to HT-1376, RTv4, ScaBER, EJ, T24, and 5637 human bladder cancer cell lines.

The EGFR expression level varies widely in different types of cancer cells. Therefore, binding assay was carried out with ^125^I-iodoEGF to confirm the presence of EGFR on the available bladder cell lines: ScaBER, RTv4, EJ, T24, and 5637. To determine the number of EGFR on the cell surface of the studied cell lines, the dependence of cell binding on concentrations of the labeled EGF in the medium was studied at 4°C. The number of specific binding sites and the equilibrium dissociation constant were determined as parameters of the equation describing the equilibrium reversible interaction of a monovalent ligand with a monovalent receptor. The experiments showed that all these cell lines have noticeable expression of EGFR (Figure 1 and Table 1).

**Table 1:** Radioligand assay of human bladder cancer cells.

Cell line	B_max_, sites per cell, mean ± SEM	K_d_, nM
HT-1376	211000 ± 22000	10.3 ± 2.4
RTv4	170000 ± 5800	2.4 ± 0.25
ScaBER	169000 ± 8800	5.8 ± 0.8
EJ	162000 ± 9200	3.8 ± 1.0
T24	40000 ± 2200	3.4 ± 0.4
5637	33000 ± 9800	2.4 ± 2.2


*B_max_, quantity of specific binding sites; K_d_, equilibrium dissociation constant of ^125^I-iodoEGF–receptor complex.*

### Cytotoxicity Studies

The EGFR-targeted MNT with covalently attached NOTA chelator was labeled with ^111^In as described in the Section “Materials and Methods.” The fraction of the isotope not attached to the MNT did not exceed 3–5% of total radioactivity as determined by denaturing Laemmli SDS-colony formation assay because this method allows estimation of the proportion of cells capable of unlimited division. It is especially important for the assessment of anticancer radiopharmaceuticals since reveals the completeness of cancer cell population eradication.

**FIGURE 2:**
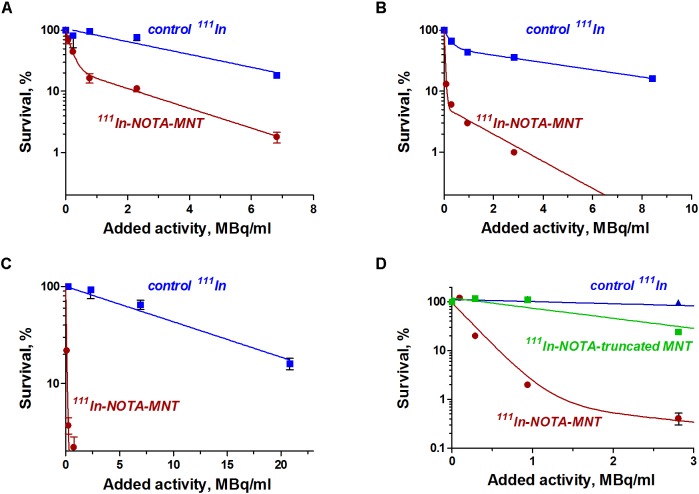
Cytotoxic efficacy of ^111^In delivered by EGFR-targeted MNTs. Cytotoxicity of ^111^In-NOTA-MNT on **(A)** 5637, **(B)** EJ, and **(C)** HT-1376 human bladder cancer cell lines compared to control ^111^In. **(D)** Cytotoxicity of ^111^In attached to EGFR-targeted NOTA-MNT compared to ^111^In attached to truncated non-targeted NOTA-MNT on EJ human bladder cancer cell line. The solid lines represent a fit of the data to either a mono- or two-exponential model. Error bars represent standard errors of mean (*n* = 3–6).

The dependence of the cytotoxic effect of free control indium-111 on its concentration for the investigated bladder cancer cell lines was approximated well by the single-exponential equation model. A different picture was observed for the cytotoxic action of ^111^In-NOTA-DTox-HMP-NLS-EGF. In most cases, these dependences were better fitted to the two-exponential equation model. This type of dependence of cell survival on radioactivity concentration of the added substance can indicate heterogeneity of the cell population in relation to the MNT-delivered ^111^In action. All investigated bladder cancer cell lines demonstrate significant enhancement of ^111^In cytotoxicity after its attachment to EGFR-targeted MNT (Figures 2A–C and Table 2). Even though the number of EGFR per cell for the bladder cancer cell lines was at least an order of magnitude lower than the extremely high EGFR expression level in A431 human epidermoid carcinoma line (∼2.5 × 10^6^ receptors per cell), the effectiveness of MNT in increasing the cytotoxic effect was rather pronounced (20–70 times compared to the free ^111^In). The increase in cytotoxicity of MNT was strictly related to the presence of the EGF ligand within the MNT structure, since ^111^In attached to the truncated MNT lacking any ligand module had only minor impact on cytotoxic effect when compared with free ^111^In (Figure 2D).

**Table 2:** Cytotoxicity of ^111^In-NOTA-DTox-HMP-NLS-EGF and free ^111^In on cancer cell lines.

Cell line	A_37_, MBq/ml		Enhancement of cytotoxicity, times
	^111^In-NOTA-MNT	^111^In	
A431	0.023	6.3	271
HT1376	0.19	13.0	70
EJ	0.039	2.22	57
5637	0.41	10.5	19


*A_37_, value of radioactivity that reduces cell survival by a factor of “e.”*

### Animal Model

**FIGURE 3:**
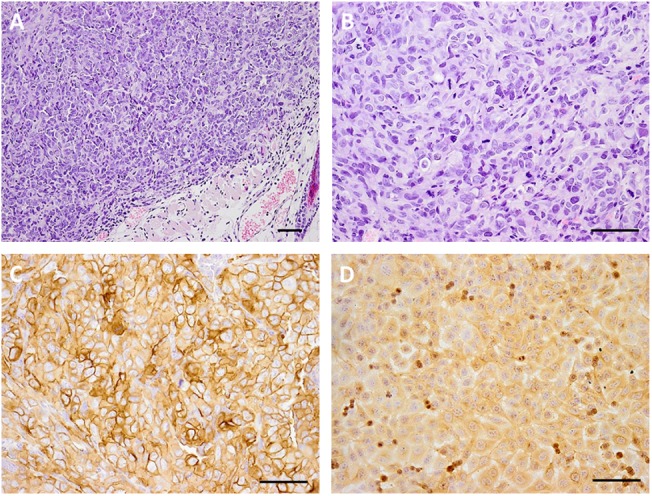
Microphotography of human bladder carcinoma EJ xenograft sections and their EGFR expression. Hematoxylin and eosin staining of paraffin-fixed sections of subcutaneous human bladder carcinoma EJ xenograft **(A)**, magnification ×100 and **(B)** magnification ×200; immunohistochemical verification of EGFR expression in **(C)** EJ xenograft sections, magnification ×200, and **(D)** 10% formalin-fixed EJ cells in culture, magnification ×200. Scale bars, 100 μm.

The EJ human bladder cancer cells were used as a model for evaluating the therapeutic efficacy and biodistribution of the EGFR-targeted MNT. This cell line has moderate EGFR expression and demonstrated a moderate increase in MNT-mediated cytotoxicity of ^111^In among the investigated cell lines. The expression of EGFR *in vivo* in the EJ tumors was confirmed by immunohistochemical studies (Figure 3). Histological examination of subcutaneous tumors showed that at the microscopic level, the EJ carcinoma xenografts grow as solid well-*vascularized* nodules surrounded by subcutaneous loose connective tissue (Figures 3A,B). Only small foci of necrosis were traced on the 40th day of xenograft growth in the central areas of the tumor tissue. Tumor metastases were not detected on this day in the regional inguinal and ipsilateral axillary lymph nodes located on the tumor side. The expression of EGFR in tumor xenograft tissue was determined using the immunoperoxidase technique, revealing intensive positive staining in all tumor cells, being slightly heterogeneous in staining pattern (Figures 3C,D). The immunoperoxidase reaction with anti-EGFR antibodies predominantly stained the surface membrane of the cells both in cultured EJ cells and in cells of the subcutaneous xenograft of EJ carcinoma. These results indicated that EJ carcinoma xenografts can be used as a suitable model for ^111^In-bearing EGFR-targeted MNT radiotherapy studies.

### SPECT/CT Imaging

**FIGURE 4:**
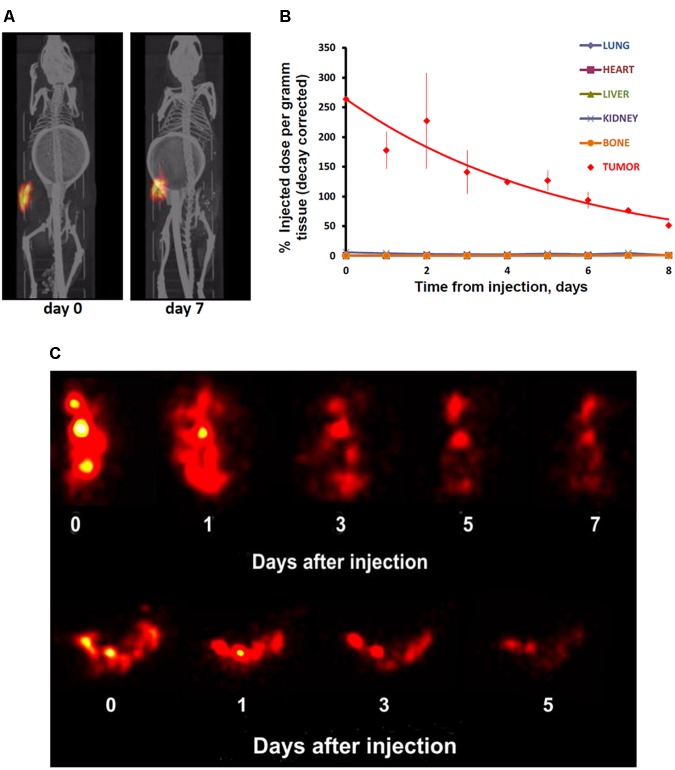
Serial SPECT/CT imaging of nude mice with subcutaneous EJ human bladder carcinoma xenografts after intratumoral injection of ^111^In attached to EGFR-targeted ^111^In-NOTA-MNT. **(A)** Whole animal SPECT/CT (color/gray) images of a representative animal obtained at indicated times after injection of ^111^In-NOTA-MNT using a 1-mm-diameter pinhole collimator. Both images were individually scaled to the maximum brightness intensity. **(B)** Decay-corrected retention of ^111^In radioactivity in EJ xenograft and normal organs after EGFR-targeted ^111^In-NOTA-MNT injection. The solid line represents a fit of the data to a mono-exponential model. **(C)** High-resolution tumor SPECT images of EJ xenograft of a representative animal obtained at indicated times after injection of ^111^In-NOTA-MNT using a 0.35-mm-diameter pinhole collimator. The ring in the abdominal region presented on the CT scans of the mice is the breathing sensor.

In contrast to the free control ^111^In, ^111^In attached to EGFR-targeted MNT remained in the injection site for a long time. Following intratumoral injection, ^111^In-NOTA-DTox-HMP-NLS-EGF demonstrated good intratumoral retention of ^111^In activity (Figure 4A). The decay-corrected retention half-life from the EJ xenografts was calculated to be 4.1 ± 0.5 days according to a single exponential equation model fitting (Figure 4B). Detection of radioactivity following intratumoral administration of ^111^In-NOTA-DTox-HMP-NLS-EGF in normal tissues was quite low and limited to nearly undetectable signal observed mainly in kidneys, liver, and lymph nodes. Serial high-resolution SPECT/CT imaging (0.35 mm-diameter pinhole collimator) of the tumor revealed that radioactivity distribution within the tumor remained nearly the same for the whole period of observation (6 days) (Figure 4C).

### Pharmacokinetics and Biodistribution of ^111^In-NOTA-DTox-HMP-NLS-EGF After Intratumoral Administration

The ^111^In radioactivity in EJ xenografts as well as in blood and internal organs was studied for 5 days to characterize the distribution of ^111^In-NOTA-DTox-HMP-NLS-EGF after intratumoral injection (Table 3). The autopsy data also demonstrated long-time retention of ^111^In-labeled EGFR-targeted MNT. Redistribution of radioactivity into other organs and tissues was rather low apart from redistribution to regional lymph nodes. The highest activity of ^111^In was registered in tumor tissue during the whole period of observation. The radioactivity in tumors was 67.5 ± 1.2% of the administered dose on the 1st day after the labeled MNT injection, 44.4 ± 10.2% on the 2nd day, and 18.3 ± 6.5% on the 5th day after the administration.

**Table 3:** ^111^In radioactivity in mouse tissues after intratumoral injection of ^111^In-NOTA-DTox-HMP-NLS-EGF into EJ human bladder cancer xenografts (percent ID per gram of tissue, mean ± standard deviation).

Time Tissue	5 min	1 h	3 h	1 day	2 days	5 days
Blood	0.244 ± 0.046	0.318 ± 0.076	0.167 ± 0.010	0.062 ± 0.017	0.025 ± 0.001	0.017 ± 0.006
Tumor	367 ± 101	370 ± 176	463 ± 123	566 ± 77	264 ± 79	80 ± 63
PT skin	5.9 ± 7.8	46.9 ± 12.4	50.4 ± 24.7	58.0 ± 23.9	61.7 ± 4.0	49.0 ± 19.0
RLN	40.5 ± 38.6	45.9 ± 57.1	73.6 ± 1.5	71.0 ± 7.3	67.0 ± 34.6	41.5 ± 22.4
Liver	0.70 ± 0.60	1.18 ± 0.27	1.40 ± 0.55	1.65 ± 0.52	1.54 ± 0.40	1.33 ± 0.23
Kidney	1.82 ± 0.97	13.3 ± 5.17	12.0 ± 1.12	12.1 ± 2.8	7.71 ± 1.99	3.07 ± 2.37
Lung	0.30 ± 0.14	0.23 ± 0.10	0.135 ± 0.017	0.094 ± 0.004	0.063 ± 0.031	0.037 ± 0.016
Spleen	0.25 ± 0.18	0.280 ± 0.092	0.43 ± 0.11	0.62 ± 0.28	0.57 ± 0.22	0.52 ± 0.13
Bones	0.198 ± 0.028	1.19 ± 0.36	0.44 ± 0.18	0.578 ± 0.071	0.55 ± 0.47	0.43 ± 0.31
Bladder	0.260 ± 0.13	0.196 ± 0.073	0.253 ± 0.083	0.074 ± 0.013	0.051 ± 0.038	0.041 ± 0.021
Stomach	0.29 ± 0.10	0.35 ± 0.23	0.213 ± 0.008	0.140 ± 0.076	0.080 ± 0.031	0.075 ± 0.052
Intestine	0.134 ± 0.041	0.203 ± 0.098	0.255 ± 0.092	0.125 ± 0.017	0.093 ± 0.012	0.053 ± 0.043
Heart	0.21 ± 0.13	0.155 ± 0.076	0.083 ± 0.006	0.090 ± 0.026	0.059 ± 0.008	0.023 ± 0.012
Muscles	0.32 ± 0.29	0.34 ± 0.23	0.246 ± 0.078	0.206 ± 0.064	0.20 ± 0.15	0.168 ± 0.045
Skin	0.079 ± 0.019	0.610 ± 0.057	0.180 ± 0.071	0.325 ± 0.049	0.205 ± 0.021	0.225 ± 0.064


*PT, peritumoral skin samples; RLN, regional lymph node.*

Measurements of the skin adjacent to the tumor areas revealed partial redistribution of ^111^In radioactivity from the site of administration. Thus, 5 min after the intratumoral injection of ^111^In radioactivity, (5.9 ± 4.8)% of the injected dose per gram of tissue (ID/g) was in the skin adjacent to the tumor, and after 1 h, this value increased to (46.9 ± 12.4)% ID/g and persisted at this level up to 5 days of observation (49.0 ± 19.0% ID/g). The radioactivity in skin areas distant from the site of administration was negligible, never exceeding 0.4% ID/g for the whole observation period.

Increased radioactivity was revealed in the regional lymph nodes on the xenograft side after intratumoral injection of ^111^In-NOTA-DTox-HMP-NLS-EGF. Thus, appreciable radioactivity of ^111^In (40.5 ± 38.5% ID/g) was observed in the inguinal lymph nodes 5 min after the administration of labeled MNT, with the maximum value (73.6 ± 1.5% ID/g) reached 3 h after the administration, followed by 41.5 ± 22.4% ID/g on the 5th day of observation. Histological examination of lymph nodes from animals in this group revealed reactive changes in most of the tissue samples corresponding to sinus histiocytosis, lacking signs of metastatic lesions.

The peak ^111^In radioactivity in blood, (0.32 ± 0.08% ID/g), was observed 1 h after intratumoral administration of ^111^In-NOTA-DTox-HMP-NLS-EGF. The concentration of radioactivity in the bloodstream did not exceed 0.03% ID/g in the time interval from 2 to 5 days after the MNT infusion. The area under the curve (AUC120) was 5.75% ID/g⋅h, and the bloodstream elimination half-life was 3.2 h.

In kidneys of the mice, the maximum concentration of radioactivity was detected in the interval from 3 h to 1 day after the administration of ^111^In-NOTA-DTox-HMP-NLS-EGF and was 12.5 ± 3.0% ID/g. Later, by 5 days of observation, a decrease to 3.1–2.0% ID/g of tissue was observed. In the liver of the animals, the concentration of radioactivity staid at a level from 0.71 to 1.64% ID/g with insignificant fluctuations during all 5 days of observation.

In general, on the 1st day after intratumoral injection of labeled MNT and beyond, the concentration of radioactivity in the tumor was 1000 or more times higher than in most normal organs and tissues. The total amount of ^111^In radioactivity that was detected in the mice excluding the peritumoral region and lymph nodes was 10.3% of the administered dose on the 1st day and 5.7% of the administered dose on the 5th day after the intratumoral injection.

### Radiotherapy of Subcutaneous EJ Tumors

**FIGURE 5:**
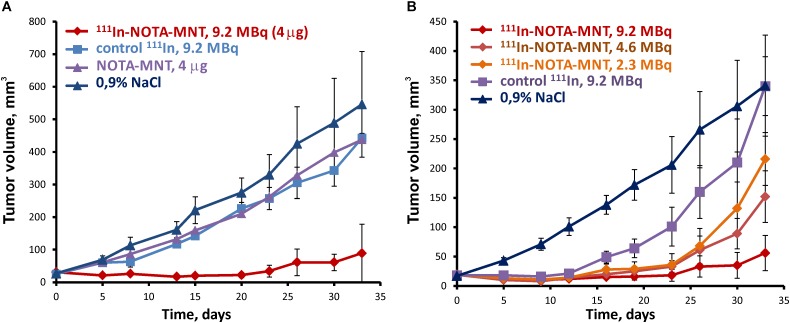
Antitumor efficacy of ^111^In delivered by the EGFR-targeted MNT on human bladder cancer xenografts in nude mice. **(A)** Tumor growth following intratumoral injection of 9.2 MBq (4 μg) of ^111^In-NOTA-MNT, 9.2 MBq of ^111^In without MNT, 4 μg NOTA-MNT without ^111^In, or saline into subcutaneous EJ xenografts. **(B)** Tumor growth following intratumoral injection of 9.2 MBq (4 μg), or 4.6 MBq (2 μg), or 2.3 MBq (1 μg) of ^111^In-NOTA-MNT into subcutaneous EJ xenografts.

To study antitumor efficacy of the ^111^In delivered by EGFR-targeted MNT on human bladder cancer xenografts, 9.2 MBq (4 μg) of ^111^In-NOTA-DTox-HMP-NLS-EGF, 9.2 MBq of ^111^In without MNT, 4 μg NOTA-DTox-HMP-NLS-EGF without ^111^In, or saline were intratumorally injected into subcutaneous EJ xenografts on the 14th day after establishing the EJ tumor. Pronounced inhibition of xenograft growth was observed after a single intratumoral injection of ^111^In-NOTA-DTox-HMP-NLS-EGF in comparison to all control groups (Figure 5A). Statistically significant differences in the mean tumor volume were observed between the group with ^111^In-NOTA-DTox-HMP-NLS-EGF injection and all control groups from the 5th day after the MNT injection. Until the 26th day after a single intratumoral injection of ^111^In-NOTA-DTox-HMP-NLS-EGF in most animals of this experimental group, the tumors did not increase in size. On the 26th day after beginning the treatment, the mean tumor volume in the group of mice receiving injection of ^111^In-NOTA-DTox-HMP-NLS-EGF (52 ± 15 mm^3^) was 9 times less than the mean tumor volume in the control group injected with saline (402 ± 78 mm^3^). A tendency to resume xenograft growth in the group of animals that were injected with ^111^In-NOTA-DTox-HMP-NLS-EGF was observed only on the 30th day after the treatment. Statistically significant differences in the mean tumor volume in the group of animals treated with labeled MNT from the initial value in the tumor before treatment were detected only on the 33rd day after the MNT injection. Injection of the same quantity of this EGFR-targeted MNT alone or ^111^In alone at the same dose did not lead to a noticeable change in tumor growth rate (Figure 5A).

Reduction of the labeled MNT dose to 4.6 and 2.3 MBq per mouse (2 and 1 μg MNT per mouse, respectively) resulted in a dose-dependent decrease in the antitumor effect (Figure 5B). At the end of this experiment, 2 of 5 mice that were injected with 9.2 MBq of ^111^In-NOTA-DTox-HMP-NLS-EGF per mouse did not have any detectable tumor.

## Discussion

The MNT DTox-HMP-NLS-EGF with human EGF as a ligand module was designed as a platform for delivering a cytotoxic agent to the nucleus of tumor cells overexpressing EGFR. We showed earlier that the attachment of short-range particle emitters ^211^At, ^125^I, ^67^Ga, or ^111^In to the MNT result in a great increase in their cytotoxicity (Rosenkranz et al., 2008; Slastnikova et al., 2012a, 2017b; Koumarianou et al., 2014), like the attachment of the photosensitizer chlorin e_6_ increased the cytotoxicity and efficacy of photodynamic therapy of human xenograft tumors in mice (Slastnikova et al., 2012b). Keeping in mind the background EGFR expression in many normal epithelial tissues, we suggest that in contrast to photodynamic therapy promoted by local light irradiation, the risks for side effects in the case of EGFR-targeted radiotherapy are substantially increased. This limits to some extent the scope of the clinical application of the approach described here; therefore, along with the well-studied MNTs created earlier (Rosenkranz et al., 2017; Slastnikova et al., 2017a,b), we are also engaged in the development of the new ones, which are designed to be better suited for systemic injections (Khramtsov et al., 2017, 2018). Nevertheless, there are some cases where the EGFR-targeted radiotherapy could be highly in demand for local administration, for instance, in the case of bladder cancer and brain malignances. The goal of this study was to estimate the antitumor efficacy of the EGFR-targeted MNT labeled with AE emitter ^111^In as well as its pharmacokinetics and biodistribution following locoregional administration. Human urinary bladder carcinoma was used as a tumor model to evaluate the specific properties of ^111^In-NOTA-DTox-HMP-NLS-EGF. Tumors of this type are often characterized by EGFR overexpression, making them suitable candidates for EGFR-targeted therapy (Neal et al., 1990; Bue et al., 1998; Chow et al., 2001; Litlekalsoy et al., 2007; Khaled et al., 2009; Carlsson et al., 2015; Mooso et al., 2015). Enhancement of EGFR expression in a tumor is often associated with poor clinical outcome (Nguyen et al., 1994; Mellon et al., 1995; Kassouf et al., 2008; Khaled et al., 2009). The normal urothelium generally lacks any noticeable EGFR expression, but the most common type of urinary bladder cancer, transitional cell carcinoma, in most cases is characterized by expression of this receptor in the superficial layer (Messing, 1990; Chow et al., 2001; Kassouf et al., 2008). Three-quarters of newly diagnosed urothelial carcinomas are non-muscle-invasive (Sexton et al., 2010), providing an opportunity for intravesically administered EGFR ligands to reach cancer cells and interact with their receptors. Moreover, recurrence rate of 50–70% of these non-muscle-invasive urothelial carcinomas (Kamat et al., 2016; Soukup et al., 2017) prompts the development of new tumor-targeted therapeutics for specific eradication of the residual cancer cells. It was demonstrated that EGF-dextran conjugate labeled with ^99m^Tc selectively accumulates in human bladder tumor tissue after instillation into the bladder before tumor resection (Bue et al., 2000). Intravesical application of targeting EGFR ^213^Bi α-particle emitter containing immunoconjugate showed promising results in early tumor stages using mouse *in situ* human bladder cancer xenografts (Pfost et al., 2009; Fazel et al., 2015). Recently, this EGFR-targeted α-emitter bearing radiopharmaceutical has been applied in a pilot study for patients with bladder carcinoma *in situ* instead of the usual cystectomy (Autenrieth et al., 2018). The AE emitters are another promising type of radionuclide. Their short-range electrons are also characterized by high linear energy transfer combined in their case with extremely short range, making them highly effective upon decay in proximity to nuclear DNA (Buchegger et al., 2006; Howell, 2008). This highly efficient local damage to sensitive biomolecules along with negligible cytotoxicity outside the cell, being the main advantages of application of AE emitters for cancer treatment, can be realized when the radionuclide is specifically transported into the nuclei of cancer cells without affecting surrounding normal cells. Like many other promising anticancer drugs and their delivery systems, MNTs exploit cell surface receptor binding and internalization to target cancer the cell; however, these transport steps ultimately lead to translocation into lysosomes rather than the cell nucleus (Rosenkranz et al., 2014; Sobolev et al., 2016). The cell nucleus can be accessed only from the cytosol; therefore, MNTs should be able to leave the endosomes prior to subsequent transport into the nucleus. Endosomal escape is provided by the endosomolytic module of the MNT, which increases its lipophilicity, inserts in phospholipid the bilayer, and forms pores in response to the decreased pH of acidifying endosomes (Khramtsov et al., 2008) to ensure penetration through the endosomal membrane. We showed earlier the efficiency of MNT DTox-HMP-NLS-EGF for its EGFR-mediated cell recognition and endocytosis and endosome escape, along with accumulation of the MNT in the cell nuclei of target cancer cells with EGFR overexpression *in vitro* (Gilyazova et al., 2006; Slastnikova et al., 2012a; Koumarianou et al., 2014) and *in vivo* (Slastnikova et al., 2012b). Theoretical comparative estimation of efficacy of various locally administered AE emitters delivered by EGFR-targeted MNT demonstrated that AE emitters are superior to the longer range α-particle emitters. Moreover, wide variability of estimated efficiency between different AE emitters themselves has been observed. Based on favorable tumor dose selectivity along with reasonable half-life and the protein labeling suitability several different AE emitters, including ^111^In and ^67^Ga already extensively used in clinic for diagnostics were suggested to warrant further investigation (Raghavan et al., 2017).

We recently developed a technique for efficient labeling of MNTs with ^111^In, producing ^111^In-MNTs with high specific activity and radiochemical purity (Slastnikova et al., 2017b), which increased cytotoxicity of ^111^In-MNTs substantially and improved potential clinical feasibility of this approach. Utilizing this labeling technique, we revealed that targeted ^111^In delivery by MNTs aimed at various tumor-specific receptors gives promising results *in vitro* and *in vivo* (Rosenkranz et al., 2017; Slastnikova et al., 2017a,b). These results encouraged us to perform a more in-depth study focused here on EGFR-targeted MNT delivery of ^111^In on the urinary bladder carcinoma model. In addition to greatly enhanced cytotoxicity and significant tumor growth inhibition, as well as prolonged tumor retention of radioactivity, which were anticipated from previous studies (Rosenkranz et al., 2017; Slastnikova et al., 2017a,b), ^111^In-labeled EGFR-targeted MNTs are demonstrated to possess promising for clinical translation pharmacokinetic profile.

^111^In emits per decay 14.7 AE of broad energy and range spectrum varying from 0.0085 keV (0.251 nm) to 22.5 keV (13600 nm) (Howell, 1992). The dependence of direct induction of DNA double-strand breaks by AE emitted by ^111^In on their energy and distance from the DNA center has been simulated recently (Piroozfar et al., 2018). With both the emission probability and efficiency of electron energy taken into consideration, 350 eV AE produced the maximum number of double-strand breaks. Though the range of 350 eV AE in water is estimated to be 16.4 nm (Howell, 1992), simulation data determined a 6-nm distance of decaying ^111^In from the DNA center as a cutoff for substantial contribution to double-strand breaks formation (Piroozfar et al., 2018). Assuming homogenous distribution of nuclear DNA [3 × 10^9^ base pairs (Ratilainen et al., 2001) resulting in approximately 2 m length of human diploid nuclear DNA in a cell], we can estimate the mean distance from any point inside the nucleus to the DNA central axis, ranging from 5.3 nm to 13.5 nm for cell nuclei diameter of 7 to 13 μm, respectively. Thus, when assuming homogeneous intranuclear distribution of ^111^In-MNT that reached the nucleus, we conclude that some proportion of the delivered ^111^In would result in considerable DNA damage, though the effect can be enhanced by further approach of ^111^In-MNT to DNA. Therefore, there is room for possible improvement, for example, by introduction of an additional DNA-binding module into the MNT.

The high local production of the low energy AE can result in not only direct action on sensitive biomolecules, but also in generation of free radicals and reactive oxygen species (Uehara and Nikjoo, 2006). These highly active products also lead to damage to molecules eliciting cell responses by activation of regulatory pathways by oxidative stress. In particular, AE emitter iodine-125 attached to non-internalizable antibodies to carcino-embryonic antigen was able to activate various signaling pathways in A431 cells expressing this antigen (Paillas et al., 2016). Although the possibility of non-specific effects of radionuclide therapy cannot be denied, it is more likely that well-designed transport into the nucleus should lead to the more prominent effect for the same AE emitter. So, the accumulated activity of ^125^I-MNT resulting in 37% survival was 3000 decays per one A431 cell (Slastnikova et al., 2012a), whereas ca. 100000 decays per the same cell were necessary to achieve the same survival rate with ^125^I-labeled non-internalizable antibody (Pouget et al., 2008).

We consider the MNTs as an upcoming flexible platform that can be adapted to different therapy options. By changing the set of functional modules in MNTs, it is possible to target the active principle into cells of different types, at different intracellular targets or adjust pharmacokinetics to better match the problems to be solved. In our conviction there is no one-stop solution to the problem of delivering drugs for cancer treatment, and each created drug vehicle has its limitations. The same fully applies to the delivered radionuclides. ^111^In should have the greatest impact in the treatment of hard-to-detect micrometastases and single cancer cells (Hindie et al., 2016; Falzone et al., 2018), whereas its effect on large tumors is less pronounced (Buchegger et al., 2006; Bomanji and Papathanasiou, 2012). The ^111^In-MNT described in this article could be highly in demand for intravesical infusion in the treatment of bladder cancer, which is characterized by a high probability of recurrence (Sfakianos et al., 2014; Kamat et al., 2016; Soukup et al., 2017). The systemic application of the ^111^In MNT requires further research and possible inclusion of additional modules into the MNT structure or modification of the existing modules.

## Conclusion

Significantly enhanced cytotoxicity and tumor growth inhibition up to complete tumor resorption, prolonged tumor retention of radioactivity, along with possible clinical translation of the pharmacokinetic profile of ^111^In attached to EGFR-targeted MNT on a human bladder cancer animal model observed in this study warrants further steps toward the development of this approach.

## Data Availability

The raw data supporting the conclusions of this manuscript will be made available by the authors, without undue reservation, to any qualified researcher.

## Author Contributions

AR, TS, GG, and AS designed and evaluated the study. RY and TK designed and evaluated the therapeutic experiments with mice. AR, TS, TK, NM, MV, VP, AA, TL, AU, YK, GG, and RY performed the study and data analysis. AR, TS, and AS wrote the manuscript. All authors contributed toward data analysis, drafting, and critically revising this report and agreed to be accountable for all aspects of the work and read and approved the final manuscript.

## Conflict of Interest Statement

The authors declare that the research was conducted in the absence of any commercial or financial relationships that could be construed as a potential conflict of interest.
